# Supplementary material for: Assessing the real-world safety of docetaxel for non-small cell lung cancer: Insights from a comprehensive analysis of FAERS data
Source: PLoS One. 2025 Sep 12;20(9):e0331979. doi: 10.1371/journal.pone.0331979 (PMC12431403; doi:10.1371/journal.pone.0331979)
Supplement: S3 Table — (DOCX) [file pone.0331979.s003.docx]

Supplementary Table 3 :

All adverse events meeting the positive signal threshold at the PT level from FAERS data

| PT | Case numbers | ROR(95%Cl) | PRR(χ2) | EBGM(EBGM05) | IC(IC025) |
| --- | --- | --- | --- | --- | --- |
| Diarrhoea | 200 | 2 ( 1.73 - 2.31 ) | 1.95 ( 91.94 ) | 1.92 ( 1.7 ) | 0.94 ( 0.73 ) |
| Pneumonia | 106 | 1.71 ( 1.41 - 2.08 ) | 1.7 ( 29.83 ) | 1.68 ( 1.42 ) | 0.74 ( 0.46 ) |
| Nausea | 106 | 1.65 ( 1.36 - 2.01 ) | 1.64 ( 25.84 ) | 1.62 ( 1.37 ) | 0.69 ( 0.41 ) |
| Fatigue | 85 | 1.47 ( 1.19 - 1.83 ) | 1.46 ( 12.34 ) | 1.45 ( 1.21 ) | 0.54 ( 0.22 ) |
| Febrile neutropenia | 85 | 2.99 ( 2.4 - 3.73 ) | 2.95 ( 104.94 ) | 2.85 ( 2.37 ) | 1.51 ( 1.19 ) |
| Vomiting | 66 | 1.36 ( 1.07 - 1.74 ) | 1.36 ( 6.16 ) | 1.35 ( 1.1 ) | 0.43 ( 0.07 ) |
| Neutropenia | 65 | 2.05 ( 1.6 - 2.63 ) | 2.04 ( 33.29 ) | 2 ( 1.62 ) | 1 ( 0.63 ) |
| Dehydration | 54 | 1.68 ( 1.28 - 2.2 ) | 1.67 ( 14.2 ) | 1.65 ( 1.31 ) | 0.72 ( 0.33 ) |
| White blood cell count decreased | 54 | 3.26 ( 2.47 - 4.29 ) | 3.23 ( 78.8 ) | 3.1 ( 2.46 ) | 1.63 ( 1.23 ) |
| General physical health deterioration | 49 | 1.94 ( 1.45 - 2.58 ) | 1.93 ( 21.21 ) | 1.89 ( 1.49 ) | 0.92 ( 0.5 ) |
| Leukopenia | 46 | 3.04 ( 2.26 - 4.1 ) | 3.02 ( 59.15 ) | 2.91 ( 2.27 ) | 1.54 ( 1.11 ) |
| Alopecia | 45 | 3.92 ( 2.9 - 5.32 ) | 3.89 ( 90.56 ) | 3.7 ( 2.87 ) | 1.89 ( 1.45 ) |
| Stomatitis | 36 | 2.16 ( 1.54 - 3.01 ) | 2.15 ( 21.29 ) | 2.1 ( 1.59 ) | 1.07 ( 0.59 ) |
| Neutrophil count decreased | 34 | 2.21 ( 1.57 - 3.12 ) | 2.2 ( 21.43 ) | 2.15 ( 1.61 ) | 1.11 ( 0.61 ) |
| Hypotension | 33 | 2.2 ( 1.55 - 3.12 ) | 2.19 ( 20.56 ) | 2.14 ( 1.6 ) | 1.1 ( 0.59 ) |
| Sepsis | 29 | 1.46 ( 1.01 - 2.11 ) | 1.45 ( 4.01 ) | 1.44 ( 1.06 ) | 0.53 ( -0.01 ) |
| Acute kidney injury | 28 | 1.57 ( 1.08 - 2.29 ) | 1.57 ( 5.65 ) | 1.55 ( 1.13 ) | 0.64 ( 0.09 ) |
| Haemoptysis | 28 | 1.5 ( 1.03 - 2.19 ) | 1.5 ( 4.58 ) | 1.49 ( 1.09 ) | 0.57 ( 0.03 ) |
| Cardiac arrest | 27 | 4.39 ( 2.97 - 6.51 ) | 4.37 ( 65.1 ) | 4.12 ( 2.97 ) | 2.04 ( 1.48 ) |
| Mucosal inflammation | 27 | 2.45 ( 1.66 - 3.61 ) | 2.44 ( 22.05 ) | 2.38 ( 1.72 ) | 1.25 ( 0.69 ) |
| Hyponatraemia | 25 | 1.85 ( 1.24 - 2.76 ) | 1.84 ( 9.35 ) | 1.82 ( 1.3 ) | 0.86 ( 0.28 ) |
| Haemoglobin decreased | 23 | 1.78 ( 1.18 - 2.71 ) | 1.78 ( 7.64 ) | 1.76 ( 1.24 ) | 0.81 ( 0.21 ) |
| Septic shock | 22 | 2.96 ( 1.93 - 4.56 ) | 2.95 ( 27.03 ) | 2.85 ( 1.99 ) | 1.51 ( 0.89 ) |
| Hypokalaemia | 22 | 1.91 ( 1.25 - 2.92 ) | 1.9 ( 9.14 ) | 1.87 ( 1.31 ) | 0.91 ( 0.29 ) |
| Urinary tract infection | 22 | 2.22 ( 1.45 - 3.41 ) | 2.22 ( 14.18 ) | 2.17 ( 1.52 ) | 1.12 ( 0.5 ) |
| Gamma-glutamyltransferase increased | 21 | 3.29 ( 2.11 - 5.11 ) | 3.27 ( 31.33 ) | 3.14 ( 2.17 ) | 1.65 ( 1.02 ) |
| Atrial fibrillation | 20 | 1.63 ( 1.04 - 2.55 ) | 1.63 ( 4.7 ) | 1.61 ( 1.11 ) | 0.69 ( 0.05 ) |
| Neutropenic sepsis | 19 | 3.92 ( 2.46 - 6.25 ) | 3.9 ( 38.35 ) | 3.71 ( 2.51 ) | 1.89 ( 1.22 ) |
| Hypoxia | 18 | 1.74 ( 1.09 - 2.78 ) | 1.73 ( 5.44 ) | 1.71 ( 1.16 ) | 0.78 ( 0.1 ) |
| Muscular weakness | 18 | 2.11 ( 1.32 - 3.39 ) | 2.11 ( 10.13 ) | 2.07 ( 1.39 ) | 1.05 ( 0.37 ) |
| Myalgia | 16 | 1.72 ( 1.04 - 2.83 ) | 1.72 ( 4.64 ) | 1.69 ( 1.12 ) | 0.76 ( 0.05 ) |
| Dermatitis | 15 | 4.95 ( 2.91 - 8.4 ) | 4.93 ( 43.14 ) | 4.6 ( 2.96 ) | 2.2 ( 1.45 ) |
| Cerebral infarction | 15 | 1.8 ( 1.07 - 3.01 ) | 1.8 ( 5.14 ) | 1.77 ( 1.15 ) | 0.82 ( 0.09 ) |
| Peripheral sensory neuropathy | 15 | 5.96 ( 3.49 - 10.16 ) | 5.94 ( 55.59 ) | 5.45 ( 3.49 ) | 2.45 ( 1.69 ) |
| Blood alkaline phosphatase increased | 15 | 2.3 ( 1.37 - 3.87 ) | 2.3 ( 10.58 ) | 2.25 ( 1.46 ) | 1.17 ( 0.43 ) |
| Blood bilirubin increased | 15 | 3.02 ( 1.79 - 5.09 ) | 3.01 ( 19.15 ) | 2.91 ( 1.88 ) | 1.54 ( 0.8 ) |
| Polyneuropathy | 15 | 4.77 ( 2.81 - 8.1 ) | 4.76 ( 40.98 ) | 4.46 ( 2.86 ) | 2.16 ( 1.4 ) |
| Psychological trauma | 14 | Inf ( NaN - Inf ) | Inf ( 759.65 ) | 55.26 ( 0 ) | 5.79 ( 4.73 ) |
| Pulmonary haemorrhage | 14 | 2.76 ( 1.61 - 4.72 ) | 2.75 ( 14.88 ) | 2.67 ( 1.7 ) | 1.42 ( 0.65 ) |
| Bone pain | 13 | 2.94 ( 1.68 - 5.15 ) | 2.94 ( 15.79 ) | 2.84 ( 1.78 ) | 1.51 ( 0.71 ) |
| Respiratory tract infection | 13 | 5.01 ( 2.84 - 8.86 ) | 5 ( 38.14 ) | 4.66 ( 2.9 ) | 2.22 ( 1.42 ) |
| Chronic obstructive pulmonary disease | 13 | 1.96 ( 1.12 - 3.41 ) | 1.95 ( 5.85 ) | 1.92 ( 1.21 ) | 0.94 ( 0.16 ) |
| Ischaemic stroke | 11 | 5.54 ( 2.98 - 10.31 ) | 5.53 ( 37.03 ) | 5.11 ( 3.04 ) | 2.35 ( 1.48 ) |
| Tachycardia | 11 | 1.96 ( 1.07 - 3.58 ) | 1.96 ( 4.97 ) | 1.92 ( 1.16 ) | 0.94 ( 0.09 ) |
| Hair colour changes | 11 | 28.49 ( 13.73 - 59.13 ) | 28.42 ( 191 ) | 18.99 ( 10.31 ) | 4.25 ( 3.27 ) |
| Hair disorder | 11 | 23.01 ( 11.36 - 46.6 ) | 22.96 ( 162.34 ) | 16.43 ( 9.1 ) | 4.04 ( 3.08 ) |
| Hair texture abnormal | 11 | 10.49 ( 5.5 - 20.03 ) | 10.47 ( 79.01 ) | 8.94 ( 5.21 ) | 3.16 ( 2.26 ) |
| Madarosis | 11 | 35.19 ( 16.48 - 75.18 ) | 35.11 ( 221.33 ) | 21.71 ( 11.5 ) | 4.44 ( 3.45 ) |
| Hypocalcaemia | 11 | 3.23 ( 1.76 - 5.94 ) | 3.23 ( 15.96 ) | 3.1 ( 1.86 ) | 1.63 ( 0.77 ) |
| Diverticulitis | 10 | 3.7 ( 1.95 - 7.02 ) | 3.69 ( 18.38 ) | 3.52 ( 2.06 ) | 1.82 ( 0.91 ) |
| Hypereosinophilic syndrome | 10 | 181.27 ( 49.87 - 658.91 ) | 180.86 ( 412.78 ) | 42.51 ( 14.44 ) | 5.41 ( 4.25 ) |
| Lacrimation increased | 9 | 4.37 ( 2.21 - 8.62 ) | 4.36 ( 21.58 ) | 4.11 ( 2.33 ) | 2.04 ( 1.09 ) |
| Palmar-plantar erythrodysaesthesia syndrome | 9 | 3.6 ( 1.83 - 7.06 ) | 3.59 ( 15.79 ) | 3.43 ( 1.95 ) | 1.78 ( 0.83 ) |
| Oral candidiasis | 9 | 4.75 ( 2.4 - 9.39 ) | 4.74 ( 24.44 ) | 4.44 ( 2.51 ) | 2.15 ( 1.2 ) |
| Bronchopulmonary aspergillosis | 9 | 9.06 ( 4.47 - 18.36 ) | 9.04 ( 55.2 ) | 7.89 ( 4.37 ) | 2.98 ( 2 ) |
| White blood cell count increased | 9 | 2.12 ( 1.09 - 4.12 ) | 2.11 ( 5.09 ) | 2.07 ( 1.19 ) | 1.05 ( 0.12 ) |
| Hypoglycaemia | 8 | 3.95 ( 1.93 - 8.1 ) | 3.95 ( 16.41 ) | 3.75 ( 2.05 ) | 1.91 ( 0.91 ) |
| Loss of consciousness | 8 | 2.06 ( 1.02 - 4.17 ) | 2.06 ( 4.19 ) | 2.02 ( 1.12 ) | 1.01 ( 0.03 ) |
| Lung infiltration | 8 | 2.35 ( 1.16 - 4.77 ) | 2.35 ( 5.93 ) | 2.29 ( 1.27 ) | 1.2 ( 0.21 ) |
| Pharyngitis | 8 | 13.18 ( 6.08 - 28.54 ) | 13.15 ( 72.32 ) | 10.78 ( 5.65 ) | 3.43 ( 2.37 ) |
| Hyperhidrosis | 8 | 3 ( 1.47 - 6.11 ) | 2.99 ( 10.07 ) | 2.89 ( 1.59 ) | 1.53 ( 0.54 ) |
| Sudden death | 8 | 2.09 ( 1.03 - 4.24 ) | 2.09 ( 4.36 ) | 2.05 ( 1.13 ) | 1.03 ( 0.05 ) |
| Haematocrit decreased | 8 | 2.5 ( 1.23 - 5.08 ) | 2.49 ( 6.85 ) | 2.43 ( 1.34 ) | 1.28 ( 0.29 ) |
| Candida infection | 7 | 5.21 ( 2.4 - 11.32 ) | 5.2 ( 21.69 ) | 4.84 ( 2.53 ) | 2.27 ( 1.2 ) |
| Haematemesis | 7 | 2.88 ( 1.35 - 6.16 ) | 2.88 ( 8.15 ) | 2.78 ( 1.47 ) | 1.48 ( 0.43 ) |
| Blood magnesium decreased | 7 | 5.07 ( 2.34 - 11.01 ) | 5.06 ( 20.89 ) | 4.72 ( 2.47 ) | 2.24 ( 1.17 ) |
| Therapeutic response decreased | 7 | 5.51 ( 2.53 - 12 ) | 5.5 ( 23.43 ) | 5.09 ( 2.65 ) | 2.35 ( 1.27 ) |
| Unresponsive to stimuli | 7 | 5.94 ( 2.72 - 12.97 ) | 5.93 ( 25.9 ) | 5.45 ( 2.83 ) | 2.45 ( 1.37 ) |
| Cerebral haemorrhage | 7 | 2.16 ( 1.01 - 4.6 ) | 2.16 ( 4.19 ) | 2.11 ( 1.12 ) | 1.08 ( 0.04 ) |
| Electrocardiogram st segment elevation | 7 | 23.77 ( 9.78 - 57.82 ) | 23.74 ( 106.07 ) | 16.82 ( 7.99 ) | 4.07 ( 2.89 ) |
| Nail disorder | 6 | 2.43 ( 1.07 - 5.51 ) | 2.43 ( 4.83 ) | 2.37 ( 1.19 ) | 1.24 ( 0.13 ) |
| Respiratory arrest | 6 | 4.13 ( 1.8 - 9.47 ) | 4.12 ( 13.18 ) | 3.9 ( 1.95 ) | 1.96 ( 0.83 ) |
| Clostridium difficile colitis | 6 | 4.13 ( 1.8 - 9.47 ) | 4.12 ( 13.18 ) | 3.9 ( 1.95 ) | 1.96 ( 0.83 ) |
| Clostridium difficile infection | 6 | 5.26 ( 2.27 - 12.16 ) | 5.25 ( 18.83 ) | 4.88 ( 2.42 ) | 2.29 ( 1.14 ) |
| Traumatic lung injury | 6 | 14.82 ( 6 - 36.56 ) | 14.8 ( 60.66 ) | 11.84 ( 5.56 ) | 3.57 ( 2.35 ) |
| Myopathy | 6 | 5.34 ( 2.31 - 12.37 ) | 5.34 ( 19.26 ) | 4.95 ( 2.45 ) | 2.31 ( 1.16 ) |
| Obstruction gastric | 6 | 32.6 ( 11.84 - 89.73 ) | 32.55 ( 114.7 ) | 20.72 ( 8.88 ) | 4.37 ( 3.08 ) |
| Coronary artery disease | 6 | 5.52 ( 2.38 - 12.8 ) | 5.52 ( 20.15 ) | 5.1 ( 2.52 ) | 2.35 ( 1.2 ) |
| Inappropriate antidiuretic hormone secretion | 6 | 3.26 ( 1.43 - 7.43 ) | 3.26 ( 8.85 ) | 3.13 ( 1.57 ) | 1.65 ( 0.52 ) |
| Paraparesis | 6 | 108.66 ( 27.17 - 434.64 ) | 108.51 ( 213.06 ) | 36.84 ( 11.55 ) | 5.2 ( 3.78 ) |
| Supraventricular tachycardia | 5 | 2.69 ( 1.09 - 6.6 ) | 2.69 ( 5.04 ) | 2.61 ( 1.23 ) | 1.38 ( 0.17 ) |
| Dacryostenosis acquired | 5 | 18.11 ( 6.58 - 49.84 ) | 18.09 ( 60.53 ) | 13.81 ( 5.92 ) | 3.79 ( 2.45 ) |
| Cardiac failure acute | 5 | 2.98 ( 1.21 - 7.34 ) | 2.98 ( 6.24 ) | 2.88 ( 1.35 ) | 1.53 ( 0.31 ) |
| Neutropenic infection | 5 | 14.29 ( 5.33 - 38.3 ) | 14.28 ( 48.88 ) | 11.51 ( 5.05 ) | 3.53 ( 2.21 ) |
| Skin fissures | 5 | 2.45 ( 1 - 5.99 ) | 2.44 ( 4.08 ) | 2.38 ( 1.12 ) | 1.25 ( 0.04 ) |
| Intestinal haemorrhage | 5 | 13.58 ( 5.09 - 36.2 ) | 13.56 ( 46.56 ) | 11.05 ( 4.87 ) | 3.47 ( 2.16 ) |
| Eczema | 5 | 2.47 ( 1.01 - 6.05 ) | 2.47 ( 4.17 ) | 2.4 ( 1.13 ) | 1.26 ( 0.06 ) |
| Venous thrombosis | 5 | 4.45 ( 1.79 - 11.08 ) | 4.45 ( 12.35 ) | 4.19 ( 1.95 ) | 2.07 ( 0.84 ) |
| Osteonecrosis of jaw | 5 | 3.77 ( 1.52 - 9.34 ) | 3.77 ( 9.51 ) | 3.59 ( 1.68 ) | 1.84 ( 0.62 ) |
| Neutrophil count increased | 5 | 4.76 ( 1.91 - 11.89 ) | 4.76 ( 13.65 ) | 4.46 ( 2.07 ) | 2.16 ( 0.92 ) |
| Hypoacusis | 5 | 4.18 ( 1.68 - 10.38 ) | 4.17 ( 11.21 ) | 3.95 ( 1.84 ) | 1.98 ( 0.75 ) |
| Respiratory rate increased | 5 | 6.32 ( 2.5 - 15.95 ) | 6.31 ( 20.01 ) | 5.76 ( 2.65 ) | 2.53 ( 1.28 ) |
| Tachypnoea | 5 | 4.24 ( 1.71 - 10.55 ) | 4.24 ( 11.48 ) | 4 ( 1.87 ) | 2 ( 0.77 ) |
| Urinary tract infection bacterial | 5 | 22.63 ( 7.97 - 64.27 ) | 22.61 ( 72.89 ) | 16.25 ( 6.79 ) | 4.02 ( 2.66 ) |
| Hepatomegaly | 5 | 7.76 ( 3.04 - 19.81 ) | 7.75 ( 25.73 ) | 6.91 ( 3.15 ) | 2.79 ( 1.53 ) |
| Fluid retention | 4 | 2.71 ( 0.99 - 7.41 ) | 2.71 ( 4.12 ) | 2.63 ( 1.14 ) | 1.4 ( 0.07 ) |
| Device related infection | 4 | 2.75 ( 1.01 - 7.51 ) | 2.75 ( 4.23 ) | 2.66 ( 1.15 ) | 1.41 ( 0.08 ) |
| Superior vena cava syndrome | 4 | 4.1 ( 1.48 - 11.33 ) | 4.09 ( 8.7 ) | 3.88 ( 1.66 ) | 1.96 ( 0.61 ) |
| Oesophageal fistula | 4 | 7.49 ( 2.63 - 21.31 ) | 7.48 ( 19.75 ) | 6.7 ( 2.79 ) | 2.74 ( 1.36 ) |
| Bronchopleural fistula | 4 | 5.72 ( 2.04 - 16.02 ) | 5.71 ( 14.07 ) | 5.26 ( 2.22 ) | 2.4 ( 1.03 ) |
| Orthostatic intolerance | 4 | 108.61 ( 19.89 - 593.17 ) | 108.51 ( 142.04 ) | 36.84 ( 8.9 ) | 5.2 ( 3.51 ) |
| Onycholysis | 4 | 12.07 ( 4.08 - 35.67 ) | 12.06 ( 33.19 ) | 10.05 ( 4.06 ) | 3.33 ( 1.9 ) |
| Retching | 4 | 5.72 ( 2.04 - 16.02 ) | 5.71 ( 14.07 ) | 5.26 ( 2.22 ) | 2.4 ( 1.03 ) |
| Duodenal ulcer haemorrhage | 4 | 7.76 ( 2.72 - 22.13 ) | 7.75 ( 20.58 ) | 6.91 ( 2.87 ) | 2.79 ( 1.4 ) |
| Chylothorax | 4 | 10.86 ( 3.71 - 31.79 ) | 10.85 ( 29.82 ) | 9.21 ( 3.75 ) | 3.2 ( 1.79 ) |
| Folliculitis | 4 | 4.62 ( 1.66 - 12.83 ) | 4.62 ( 10.45 ) | 4.33 ( 1.84 ) | 2.12 ( 0.76 ) |
| Red cell distribution width increased | 4 | 14.48 ( 4.8 - 43.65 ) | 14.47 ( 39.6 ) | 11.63 ( 4.62 ) | 3.54 ( 2.1 ) |
| Pneumonia salmonella | 4 | 217.23 ( 24.27 - 1944 ) | 217.03 ( 172.03 ) | 44.21 ( 7.06 ) | 5.47 ( 3.72 ) |
| Lung abscess | 4 | 2.93 ( 1.07 - 8.03 ) | 2.93 ( 4.84 ) | 2.83 ( 1.22 ) | 1.5 ( 0.17 ) |
| Salmonellosis | 4 | 36.2 ( 10.21 - 128.34 ) | 36.17 ( 82.08 ) | 22.1 ( 7.67 ) | 4.47 ( 2.91 ) |
| Salmonella sepsis | 4 | 72.41 ( 16.2 - 323.63 ) | 72.34 ( 120.62 ) | 31.58 ( 9.02 ) | 4.98 ( 3.34 ) |
| Thrombotic thrombocytopenic purpura | 3 | 3.79 ( 1.17 - 12.21 ) | 3.79 ( 5.75 ) | 3.6 ( 1.35 ) | 1.85 ( 0.35 ) |
| Renal cancer metastatic | 3 | 162.88 ( 16.94 - 1566.27 ) | 162.77 ( 120.59 ) | 41.44 ( 6.24 ) | 5.37 ( 3.44 ) |
| Emotional distress | 3 | 12.53 ( 3.57 - 43.98 ) | 12.52 ( 25.84 ) | 10.36 ( 3.62 ) | 3.37 ( 1.77 ) |
| Abscess | 3 | 4.4 ( 1.36 - 14.28 ) | 4.4 ( 7.29 ) | 4.14 ( 1.55 ) | 2.05 ( 0.54 ) |
| Diffuse large b-cell lymphoma | 3 | 7.76 ( 2.31 - 26.01 ) | 7.75 ( 15.44 ) | 6.91 ( 2.51 ) | 2.79 ( 1.23 ) |
| Tracheo-oesophageal fistula | 3 | 5.43 ( 1.66 - 17.8 ) | 5.43 ( 9.85 ) | 5.02 ( 1.86 ) | 2.33 ( 0.8 ) |
| Metastases to skin | 3 | 8.57 ( 2.54 - 28.98 ) | 8.57 ( 17.32 ) | 7.54 ( 2.72 ) | 2.91 ( 1.35 ) |
| Bundle branch block left | 3 | 5.62 ( 1.71 - 18.44 ) | 5.61 ( 10.31 ) | 5.18 ( 1.92 ) | 2.37 ( 0.84 ) |
| Cardiac dysfunction | 3 | 3.62 ( 1.12 - 11.65 ) | 3.62 ( 5.33 ) | 3.45 ( 1.3 ) | 1.79 ( 0.29 ) |
| Cardiac failure chronic | 3 | 6.03 ( 1.83 - 19.89 ) | 6.03 ( 11.33 ) | 5.53 ( 2.04 ) | 2.47 ( 0.93 ) |
| Mitral valve incompetence | 3 | 4.07 ( 1.26 - 13.17 ) | 4.07 ( 6.46 ) | 3.86 ( 1.44 ) | 1.95 ( 0.44 ) |
| Acute myeloid leukaemia | 3 | 3.7 ( 1.15 - 11.92 ) | 3.7 ( 5.53 ) | 3.53 ( 1.33 ) | 1.82 ( 0.32 ) |
| Cell-mediated immune deficiency | 3 | 32.58 ( 7.78 - 136.36 ) | 32.55 ( 57.35 ) | 20.72 ( 6.25 ) | 4.37 ( 2.64 ) |
| Blood osmolarity decreased | 3 | 23.27 ( 6.01 - 90.01 ) | 23.25 ( 44.72 ) | 16.58 ( 5.34 ) | 4.05 ( 2.37 ) |
| Erectile dysfunction | 3 | 20.36 ( 5.4 - 76.77 ) | 20.35 ( 40.14 ) | 15.07 ( 4.96 ) | 3.91 ( 2.25 ) |
| Hypercoagulation | 3 | 11.63 ( 3.34 - 40.5 ) | 11.63 ( 24 ) | 9.75 ( 3.43 ) | 3.29 ( 1.69 ) |
| Aortic aneurysm | 3 | 7.08 ( 2.13 - 23.59 ) | 7.08 ( 13.85 ) | 6.38 ( 2.33 ) | 2.67 ( 1.13 ) |
| Immunosuppression | 3 | 23.27 ( 6.01 - 90.01 ) | 23.25 ( 44.72 ) | 16.58 ( 5.34 ) | 4.05 ( 2.37 ) |
| Hypervolaemia | 3 | 27.15 ( 6.79 - 108.58 ) | 27.13 ( 50.33 ) | 18.42 ( 5.77 ) | 4.2 ( 2.5 ) |
| Post procedural complication | 3 | 4.65 ( 1.43 - 15.14 ) | 4.65 ( 7.92 ) | 4.36 ( 1.63 ) | 2.13 ( 0.61 ) |

Abbreviation: ROR, reporting odds ratio; PRR, proportional reporting ratio; EBGM, empirical Bayesian geometric mean; EBGM05, the lower limit of the 95% CI of EBGM; IC, information component; IC025, the lower limit of the 95% CI of the IC; CI, confidence interval; PT,preferred term.
