# Supplementary material for: Assessing the real-world safety of docetaxel for non-small cell lung cancer: Insights from a comprehensive analysis of FAERS data
Source: PLoS One. 2025 Sep 12;20(9):e0331979. doi: 10.1371/journal.pone.0331979 (PMC12431403; doi:10.1371/journal.pone.0331979)
Supplement: S4 Table — (DOCX) [file pone.0331979.s004.docx]

Supplementary Table 4:

Top 50 most frequent adverse events for Docetaxel at the preferred term (PT) level in males from FAERS data

| PT | Case numbers | ROR(95%Cl) | PRR(χ2) | EBGM(EBGM05) | IC(IC025) |
| --- | --- | --- | --- | --- | --- |
| Diarrhoea* | 100 | 4.41 ( 3.61 - 5.39 ) | 4.26 ( 251.91 ) | 4.26 ( 3.6 ) | 2.09 ( 1.8 ) |
| Malignant neoplasm progression* | 74 | 14.97 ( 11.87 - 18.87 ) | 14.52 ( 931.66 ) | 14.49 ( 11.94 ) | 3.86 ( 3.52 ) |
| Pneumonia* | 73 | 5.22 ( 4.13 - 6.59 ) | 5.08 ( 240.75 ) | 5.08 ( 4.18 ) | 2.34 ( 2 ) |
| Dyspnoea* | 50 | 2.3 ( 1.74 - 3.05 ) | 2.27 ( 36.01 ) | 2.27 ( 1.8 ) | 1.18 ( 0.78 ) |
| Febrile neutropenia* | 49 | 15.92 ( 11.99 - 21.13 ) | 15.6 ( 668.97 ) | 15.57 ( 12.28 ) | 3.96 ( 3.55 ) |
| Pyrexia* | 47 | 3.08 ( 2.31 - 4.11 ) | 3.04 ( 64.68 ) | 3.04 ( 2.39 ) | 1.6 ( 1.18 ) |
| Fatigue* | 45 | 1.66 ( 1.23 - 2.23 ) | 1.64 ( 11.49 ) | 1.64 ( 1.28 ) | 0.72 ( 0.29 ) |
| Nausea* | 41 | 1.84 ( 1.35 - 2.5 ) | 1.82 ( 15.36 ) | 1.82 ( 1.41 ) | 0.87 ( 0.42 ) |
| Neutropenia* | 40 | 7.56 ( 5.53 - 10.34 ) | 7.45 ( 223.62 ) | 7.44 ( 5.73 ) | 2.9 ( 2.44 ) |
| Death | 39 | 0.89 ( 0.65 - 1.23 ) | 0.9 ( 0.48 ) | 0.9 ( 0.69 ) | -0.16 ( -0.62 ) |
| General physical health deterioration* | 33 | 6.81 ( 4.83 - 9.6 ) | 6.72 ( 161.01 ) | 6.72 ( 5.04 ) | 2.75 ( 2.25 ) |
| Leukopenia* | 32 | 15.47 ( 10.91 - 21.94 ) | 15.27 ( 426.28 ) | 15.24 ( 11.38 ) | 3.93 ( 3.42 ) |
| Dehydration* | 31 | 5.34 ( 3.75 - 7.61 ) | 5.28 ( 107.8 ) | 5.28 ( 3.92 ) | 2.4 ( 1.89 ) |
| Asthenia* | 28 | 1.82 ( 1.25 - 2.64 ) | 1.81 ( 10.22 ) | 1.81 ( 1.33 ) | 0.86 ( 0.32 ) |
| Non-small cell lung cancer* | 28 | 122.25 ( 83.97 - 177.99 ) | 120.77 ( 3273.5 ) | 118.87 ( 86.81 ) | 6.89 ( 6.35 ) |
| White blood cell count decreased* | 27 | 6.52 ( 4.46 - 9.53 ) | 6.45 ( 124.51 ) | 6.45 ( 4.69 ) | 2.69 ( 2.14 ) |
| Hypotension* | 26 | 2.74 ( 1.86 - 4.03 ) | 2.72 ( 28.35 ) | 2.72 ( 1.97 ) | 1.44 ( 0.88 ) |
| Neutrophil count decreased* | 26 | 14.8 ( 10.05 - 21.79 ) | 14.64 ( 330.1 ) | 14.62 ( 10.57 ) | 3.87 ( 3.31 ) |
| Vomiting* | 25 | 1.69 ( 1.14 - 2.51 ) | 1.68 ( 6.97 ) | 1.68 ( 1.21 ) | 0.75 ( 0.18 ) |
| Respiratory failure* | 24 | 6.11 ( 4.09 - 9.14 ) | 6.06 ( 101.45 ) | 6.05 ( 4.32 ) | 2.6 ( 2.02 ) |
| Decreased appetite* | 23 | 2.38 ( 1.58 - 3.58 ) | 2.36 ( 18.13 ) | 2.36 ( 1.67 ) | 1.24 ( 0.65 ) |
| Sepsis* | 22 | 3.95 ( 2.59 - 6.01 ) | 3.92 ( 47.89 ) | 3.92 ( 2.76 ) | 1.97 ( 1.36 ) |
| Pneumonitis* | 20 | 17.23 ( 11.09 - 26.77 ) | 17.09 ( 302.36 ) | 17.05 ( 11.79 ) | 4.09 ( 3.46 ) |
| Anaemia* | 19 | 2.29 ( 1.46 - 3.6 ) | 2.28 ( 13.74 ) | 2.28 ( 1.56 ) | 1.19 ( 0.54 ) |
| Disease progression* | 18 | 3.44 ( 2.16 - 5.47 ) | 3.42 ( 30.91 ) | 3.42 ( 2.32 ) | 1.77 ( 1.11 ) |
| Pleural effusion* | 18 | 5.91 ( 3.71 - 9.4 ) | 5.87 ( 72.76 ) | 5.87 ( 3.98 ) | 2.55 ( 1.89 ) |
| Septic shock* | 17 | 7.61 ( 4.72 - 12.27 ) | 7.57 ( 96.85 ) | 7.56 ( 5.07 ) | 2.92 ( 2.23 ) |
| Stomatitis* | 16 | 8.33 ( 5.09 - 13.62 ) | 8.28 ( 102.37 ) | 8.27 ( 5.48 ) | 3.05 ( 2.35 ) |
| Hypoxia* | 15 | 8.64 ( 5.2 - 14.37 ) | 8.59 ( 100.61 ) | 8.59 ( 5.61 ) | 3.1 ( 2.38 ) |
| Acute kidney injury* | 15 | 2.02 ( 1.22 - 3.36 ) | 2.02 ( 7.71 ) | 2.02 ( 1.32 ) | 1.01 ( 0.29 ) |
| Neutropenic sepsis* | 14 | 39.79 ( 23.5 - 67.38 ) | 39.55 ( 523.42 ) | 39.35 ( 25.32 ) | 5.3 ( 4.55 ) |
| Mucosal inflammation | 14 | 12.88 ( 7.61 - 21.79 ) | 12.81 ( 152.21 ) | 12.79 ( 8.24 ) | 3.68 ( 2.93 ) |
| Neoplasm progression* | 14 | 10.99 ( 6.5 - 18.59 ) | 10.93 ( 126.16 ) | 10.91 ( 7.03 ) | 3.45 ( 2.7 ) |
| Drug ineffective | 13 | 0.27 ( 0.16 - 0.47 ) | 0.28 ( 24.74 ) | 0.28 ( 0.18 ) | -1.84 ( -2.62 ) |
| Cardiac arrest* | 12 | 2.62 ( 1.49 - 4.62 ) | 2.61 ( 11.98 ) | 2.61 ( 1.63 ) | 1.39 ( 0.58 ) |
| Abdominal pain | 12 | 1.49 ( 0.85 - 2.63 ) | 1.49 ( 1.94 ) | 1.49 ( 0.93 ) | 0.58 ( -0.23 ) |
| Dysphagia* | 12 | 3.08 ( 1.75 - 5.44 ) | 3.07 ( 16.81 ) | 3.07 ( 1.91 ) | 1.62 ( 0.82 ) |
| Cough | 11 | 1.12 ( 0.62 - 2.03 ) | 1.12 ( 0.15 ) | 1.12 ( 0.68 ) | 0.17 ( -0.67 ) |
| Myalgia | 11 | 1.7 ( 0.94 - 3.08 ) | 1.7 ( 3.19 ) | 1.7 ( 1.04 ) | 0.77 ( -0.07 ) |
| Interstitial lung disease* | 11 | 4.39 ( 2.43 - 7.93 ) | 4.37 ( 28.6 ) | 4.37 ( 2.66 ) | 2.13 ( 1.29 ) |
| Pulmonary embolism* | 11 | 2.83 ( 1.56 - 5.12 ) | 2.82 ( 12.94 ) | 2.82 ( 1.72 ) | 1.5 ( 0.66 ) |
| Haemoglobin decreased* | 11 | 2.21 ( 1.22 - 4 ) | 2.21 ( 7.27 ) | 2.21 ( 1.34 ) | 1.14 ( 0.31 ) |
| Confusional state | 11 | 1.5 ( 0.83 - 2.71 ) | 1.5 ( 1.82 ) | 1.5 ( 0.91 ) | 0.58 ( -0.25 ) |
| Hyponatraemia* | 10 | 4.29 ( 2.31 - 7.99 ) | 4.28 ( 25.13 ) | 4.28 ( 2.54 ) | 2.1 ( 1.22 ) |
| Respiratory tract infection* | 10 | 11.6 ( 6.23 - 21.61 ) | 11.56 ( 96.32 ) | 11.54 ( 6.86 ) | 3.53 ( 2.66 ) |
| Atrial fibrillation* | 10 | 1.94 ( 1.04 - 3.61 ) | 1.94 ( 4.54 ) | 1.94 ( 1.15 ) | 0.95 ( 0.08 ) |
| Haemoptysis* | 10 | 6.21 ( 3.33 - 11.55 ) | 6.18 ( 43.45 ) | 6.18 ( 3.67 ) | 2.63 ( 1.75 ) |
| Chronic obstructive pulmonary disease* | 10 | 4.28 ( 2.3 - 7.97 ) | 4.27 ( 25.03 ) | 4.27 ( 2.54 ) | 2.09 ( 1.22 ) |
| Pneumothorax* | 9 | 10.46 ( 5.43 - 20.14 ) | 10.42 ( 76.6 ) | 10.41 ( 6.02 ) | 3.38 ( 2.46 ) |
| Condition aggravated | 9 | 0.87 ( 0.45 - 1.68 ) | 0.87 ( 0.16 ) | 0.87 ( 0.51 ) | -0.19 ( -1.11 ) |

Abbreviation: Asterisks (*) indicate statistically significant signals in algorithm; ROR, reporting odds ratio; PRR, proportional reporting ratio; EBGM, empirical Bayesian geometric mean; EBGM05, the lower limit of the 95% CI of EBGM; IC, information component; IC025, the lower limit of the 95% CI of the IC; CI, confidence interval; PT,preferred term; AEs, adverse events.
