# Supplementary material for: Assessing the real-world safety of docetaxel for non-small cell lung cancer: Insights from a comprehensive analysis of FAERS data
Source: PLoS One. 2025 Sep 12;20(9):e0331979. doi: 10.1371/journal.pone.0331979 (PMC12431403; doi:10.1371/journal.pone.0331979)
Supplement: S5 Table — (DOCX) [file pone.0331979.s005.docx]

Supplementary Table 5:

Top 50 most frequent adverse events for Docetaxel at the PT level in females from FAERS data

| PT | Case numbers | ROR(95%Cl) | PRR(χ2) | EBGM(EBGM05) | IC(IC025) |
| --- | --- | --- | --- | --- | --- |
| Diarrhoea* | 94 | 5.15 ( 4.18 - 6.34 ) | 4.92 ( 297.03 ) | 4.92 ( 4.14 ) | 2.3 ( 2 ) |
| Nausea* | 61 | 2.28 ( 1.77 - 2.95 ) | 2.24 ( 42.53 ) | 2.24 ( 1.81 ) | 1.16 ( 0.79 ) |
| Fatigue* | 38 | 1.6 ( 1.16 - 2.21 ) | 1.59 ( 8.36 ) | 1.59 ( 1.21 ) | 0.67 ( 0.2 ) |
| Vomiting* | 38 | 2.53 ( 1.83 - 3.49 ) | 2.49 ( 34.33 ) | 2.49 ( 1.91 ) | 1.32 ( 0.85 ) |
| Alopecia* | 37 | 4.7 ( 3.39 - 6.51 ) | 4.62 ( 105.46 ) | 4.62 ( 3.52 ) | 2.21 ( 1.73 ) |
| Malignant neoplasm progression* | 28 | 12.79 ( 8.8 - 18.58 ) | 12.6 ( 299.16 ) | 12.59 ( 9.21 ) | 3.65 ( 3.11 ) |
| Anaemia* | 26 | 5.09 ( 3.45 - 7.49 ) | 5.03 ( 84.08 ) | 5.03 ( 3.63 ) | 2.33 ( 1.77 ) |
| White blood cell count decreased* | 24 | 7.24 ( 4.84 - 10.83 ) | 7.15 ( 127.14 ) | 7.15 ( 5.1 ) | 2.84 ( 2.26 ) |
| Disease progression* | 24 | 9.42 ( 6.29 - 14.09 ) | 9.3 ( 177.96 ) | 9.3 ( 6.64 ) | 3.22 ( 2.64 ) |
| Pneumonia* | 22 | 2.63 ( 1.72 - 4 ) | 2.6 ( 21.85 ) | 2.6 ( 1.83 ) | 1.38 ( 0.78 ) |
| Neutropenia* | 22 | 7.31 ( 4.8 - 11.13 ) | 7.23 ( 118.22 ) | 7.23 ( 5.08 ) | 2.85 ( 2.25 ) |
| Stomatitis* | 20 | 10.69 ( 6.88 - 16.61 ) | 10.58 ( 173.54 ) | 10.57 ( 7.31 ) | 3.4 ( 2.77 ) |
| Dehydration* | 19 | 4.9 ( 3.12 - 7.7 ) | 4.85 ( 58.27 ) | 4.85 ( 3.32 ) | 2.28 ( 1.63 ) |
| Hypokalaemia* | 19 | 14.29 ( 9.09 - 22.47 ) | 14.15 ( 232.12 ) | 14.14 ( 9.68 ) | 3.82 ( 3.17 ) |
| Gamma-glutamyltransferase increased* | 18 | 29.8 ( 18.72 - 47.43 ) | 29.5 ( 494.88 ) | 29.45 ( 19.96 ) | 4.88 ( 4.21 ) |
| Urinary tract infection* | 17 | 2.75 ( 1.71 - 4.44 ) | 2.73 ( 18.77 ) | 2.73 ( 1.83 ) | 1.45 ( 0.77 ) |
| Febrile neutropenia* | 17 | 12.81 ( 7.95 - 20.67 ) | 12.7 ( 183.23 ) | 12.69 ( 8.51 ) | 3.67 ( 2.98 ) |
| General physical health deterioration* | 16 | 5.69 ( 3.48 - 9.31 ) | 5.65 ( 61.29 ) | 5.65 ( 3.74 ) | 2.5 ( 1.79 ) |
| Death | 16 | 0.91 ( 0.55 - 1.48 ) | 0.91 ( 0.16 ) | 0.91 ( 0.6 ) | -0.14 ( -0.84 ) |
| Non-small cell lung cancer* | 16 | 174.85 ( 106.6 - 286.79 ) | 173.26 ( 2711.6 ) | 171.45 ( 113.32 ) | 7.42 ( 6.71 ) |
| Muscular weakness* | 16 | 4.55 ( 2.78 - 7.44 ) | 4.51 ( 43.83 ) | 4.51 ( 2.99 ) | 2.17 ( 1.47 ) |
| Decreased appetite* | 16 | 2.55 ( 1.56 - 4.17 ) | 2.53 ( 14.89 ) | 2.53 ( 1.68 ) | 1.34 ( 0.64 ) |
| Hyponatraemia* | 15 | 9.41 ( 5.66 - 15.65 ) | 9.34 ( 111.75 ) | 9.34 ( 6.1 ) | 3.22 ( 2.5 ) |
| Aspartate aminotransferase increased* | 14 | 9.9 ( 5.85 - 16.75 ) | 9.83 ( 111.01 ) | 9.82 ( 6.32 ) | 3.3 ( 2.55 ) |
| Blood alkaline phosphatase increased* | 14 | 19.47 ( 11.5 - 32.95 ) | 19.32 ( 243 ) | 19.3 ( 12.42 ) | 4.27 ( 3.52 ) |
| Pyrexia | 14 | 1.47 ( 0.87 - 2.48 ) | 1.46 ( 2.05 ) | 1.46 ( 0.94 ) | 0.55 ( -0.2 ) |
| Pleural effusion* | 13 | 8.19 ( 4.74 - 14.13 ) | 8.14 ( 81.4 ) | 8.13 ( 5.15 ) | 3.02 ( 2.25 ) |
| Psychological trauma* | 13 | 28.79 ( 16.68 - 49.71 ) | 28.59 ( 345.57 ) | 28.54 ( 18.07 ) | 4.83 ( 4.06 ) |
| Cardiac arrest* | 13 | 6.73 ( 3.9 - 11.62 ) | 6.69 ( 62.98 ) | 6.69 ( 4.24 ) | 2.74 ( 1.97 ) |
| Cerebral infarction* | 11 | 20.54 ( 11.35 - 37.17 ) | 20.42 ( 202.93 ) | 20.39 ( 12.41 ) | 4.35 ( 3.51 ) |
| Blood bilirubin increased* | 11 | 17.86 ( 9.87 - 32.32 ) | 17.75 ( 173.8 ) | 17.74 ( 10.8 ) | 4.15 ( 3.31 ) |
| Leukopenia* | 11 | 8.7 ( 4.81 - 15.75 ) | 8.66 ( 74.49 ) | 8.65 ( 5.27 ) | 3.11 ( 2.28 ) |
| Mucosal inflammation* | 11 | 17.8 ( 9.84 - 32.21 ) | 17.7 ( 173.14 ) | 17.68 ( 10.76 ) | 4.14 ( 3.31 ) |
| Acute kidney injury* | 10 | 3.42 ( 1.84 - 6.37 ) | 3.41 ( 17.05 ) | 3.41 ( 2.03 ) | 1.77 ( 0.9 ) |
| Off label use | 10 | 0.49 ( 0.27 - 0.92 ) | 0.5 ( 5.15 ) | 0.5 ( 0.3 ) | -1.01 ( -1.88 ) |
| Hair colour changes* | 10 | 18.2 ( 9.77 - 33.91 ) | 18.11 ( 161.48 ) | 18.09 ( 10.75 ) | 4.18 ( 3.3 ) |
| Hair disorder* | 10 | 22.24 ( 11.94 - 41.42 ) | 22.12 ( 201.41 ) | 22.09 ( 13.13 ) | 4.47 ( 3.59 ) |
| Hair texture abnormal* | 10 | 14.82 ( 7.96 - 27.6 ) | 14.74 ( 128 ) | 14.73 ( 8.75 ) | 3.88 ( 3.01 ) |
| Madarosis* | 10 | 17.91 ( 9.62 - 33.36 ) | 17.82 ( 158.59 ) | 17.8 ( 10.58 ) | 4.15 ( 3.28 ) |
| Abdominal pain | 10 | 1.35 ( 0.73 - 2.52 ) | 1.35 ( 0.92 ) | 1.35 ( 0.8 ) | 0.44 ( -0.44 ) |
| Cerebrovascular accident* | 10 | 2.18 ( 1.17 - 4.05 ) | 2.17 ( 6.32 ) | 2.17 ( 1.29 ) | 1.12 ( 0.24 ) |
| Constipation | 10 | 1.66 ( 0.89 - 3.1 ) | 1.66 ( 2.64 ) | 1.66 ( 0.99 ) | 0.73 ( -0.14 ) |
| Asthenia | 9 | 0.8 ( 0.42 - 1.55 ) | 0.81 ( 0.42 ) | 0.81 ( 0.47 ) | -0.31 ( -1.23 ) |
| Drug ineffective | 9 | 0.24 ( 0.12 - 0.45 ) | 0.24 ( 22.16 ) | 0.24 ( 0.14 ) | -2.06 ( -2.97 ) |
| Atrial fibrillation* | 9 | 3.88 ( 2.01 - 7.46 ) | 3.86 ( 19.1 ) | 3.86 ( 2.23 ) | 1.95 ( 1.03 ) |
| Hepatotoxicity* | 9 | 17.67 ( 9.18 - 34.04 ) | 17.59 ( 140.69 ) | 17.57 ( 10.15 ) | 4.14 ( 3.22 ) |
| Peripheral sensory neuropathy* | 9 | 76.78 ( 39.82 - 148.03 ) | 76.39 ( 666.52 ) | 76.03 ( 43.9 ) | 6.25 ( 5.33 ) |
| Hypocalcaemia* | 9 | 18.61 ( 9.66 - 35.84 ) | 18.52 ( 149.01 ) | 18.5 ( 10.69 ) | 4.21 ( 3.29 ) |
| Hypereosinophilic syndrome* | 9 | 839.35 ( 428.87 - 1642.72 ) | 835.04 ( 7132.64 ) | 794.46 ( 452.97 ) | 9.63 ( 8.69 ) |
| Dyspnoea | 8 | 0.46 ( 0.23 - 0.92 ) | 0.46 ( 5 ) | 0.46 ( 0.26 ) | -1.11 ( -2.07 ) |

Abbreviation: Asterisks (*) indicate statistically significant signals in algorithm; ROR, reporting odds ratio; PRR, proportional reporting ratio; EBGM, empirical Bayesian geometric mean; EBGM05, the lower limit of the 95% CI of EBGM; IC, information component; IC025, the lower limit of the 95% CI of the IC; CI, confidence interval; PT,preferred term; AEs, adverse events.
