# Supplementary material for: Assessing the real-world safety of docetaxel for non-small cell lung cancer: Insights from a comprehensive analysis of FAERS data
Source: PLoS One. 2025 Sep 12;20(9):e0331979. doi: 10.1371/journal.pone.0331979 (PMC12431403; doi:10.1371/journal.pone.0331979)
Supplement: S6 Table — (DOCX) [file pone.0331979.s006.docx]

Supplementary Table 6:

Adverse events at the PT level for Docetaxel in patients aged under 18 from FAERS data

| PT | Case numbers | ROR(95%CI) | PRR(χ2) | EBGM(EBGM05) | IC(IC025) |
| --- | --- | --- | --- | --- | --- |
| URINARY TRACT INFECTION* | 4 | 521.51 ( 156.89 - 1733.55 ) | 348.01 ( 1382.08 ) | 347.18 ( 127.07 ) | 8.44 ( 6.92 ) |
| NEUTROPENIA* | 4 | 176.3 ( 53.07 - 585.68 ) | 117.87 ( 464.45 ) | 117.77 ( 43.13 ) | 6.88 ( 5.36 ) |
| DISEASE RECURRENCE* | 1 | 115.38 ( 14.89 - 894.3 ) | 105.85 ( 103.86 ) | 105.77 ( 19.06 ) | 6.72 ( 4.53 ) |
| HERPES ZOSTER* | 1 | 269.14 ( 34.69 - 2088.02 ) | 246.8 ( 244.47 ) | 246.38 ( 44.37 ) | 7.94 ( 5.75 ) |
| MUCOSAL INFLAMMATION* | 1 | 69.53 ( 8.97 - 538.81 ) | 63.82 ( 61.89 ) | 63.79 ( 11.5 ) | 6 ( 3.8 ) |
| OESOPHAGITIS* | 1 | 585.71 ( 75.36 - 4552.44 ) | 536.99 ( 533.12 ) | 535.03 ( 96.21 ) | 9.06 ( 6.86 ) |

Abbreviation: Asterisks (*) indicate statistically significant signals in algorithm; ROR, reporting odds ratio; PRR, proportional reporting ratio; EBGM, empirical Bayesian geometric mean; EBGM05, the lower limit of the 95% CI of EBGM; IC, information component; IC025, the lower limit of the 95% CI of the IC; CI, confidence interval; PT, preferred term.
