# Supplementary material for: Assessing the real-world safety of docetaxel for non-small cell lung cancer: Insights from a comprehensive analysis of FAERS data
Source: PLoS One. 2025 Sep 12;20(9):e0331979. doi: 10.1371/journal.pone.0331979 (PMC12431403; doi:10.1371/journal.pone.0331979)
Supplement: S7 Table — (DOCX) [file pone.0331979.s007.docx]

Supplementary Table 7:

Top 50 most frequent adverse events for Docetaxel at the PT level in patients aged 18 to 65 from FAERS data

| PT | Case numbers | ROR(95%Cl) | PRR(χ2) | EBGM(EBGM05) | IC(IC025) |
| --- | --- | --- | --- | --- | --- |
| Diarrhoea* | 90 | 4.59 ( 3.71 - 5.67 ) | 4.43 ( 241.41 ) | 4.43 ( 3.71 ) | 2.15 ( 1.84 ) |
| Malignant neoplasm progression* | 65 | 24.8 ( 19.36 - 31.76 ) | 24.04 ( 1433.73 ) | 23.98 ( 19.5 ) | 4.58 ( 4.22 ) |
| Nausea* | 63 | 2.05 ( 1.59 - 2.63 ) | 2.02 ( 32.81 ) | 2.02 ( 1.64 ) | 1.01 ( 0.65 ) |
| Fatigue* | 55 | 2.01 ( 1.53 - 2.62 ) | 1.98 ( 27 ) | 1.98 ( 1.58 ) | 0.98 ( 0.59 ) |
| Vomiting* | 39 | 2.2 ( 1.6 - 3.02 ) | 2.18 ( 25.09 ) | 2.18 ( 1.67 ) | 1.12 ( 0.66 ) |
| Alopecia* | 39 | 5.4 ( 3.93 - 7.41 ) | 5.31 ( 136.91 ) | 5.31 ( 4.07 ) | 2.41 ( 1.95 ) |
| Pneumonia* | 33 | 3.69 ( 2.62 - 5.21 ) | 3.65 ( 63.65 ) | 3.65 ( 2.73 ) | 1.87 ( 1.37 ) |
| Febrile neutropenia* | 32 | 15.92 ( 11.23 - 22.58 ) | 15.69 ( 439.8 ) | 15.66 ( 11.69 ) | 3.97 ( 3.46 ) |
| General physical health deterioration* | 31 | 10.3 ( 7.22 - 14.68 ) | 10.16 ( 255.98 ) | 10.15 ( 7.54 ) | 3.34 ( 2.83 ) |
| Anaemia* | 30 | 5.22 ( 3.64 - 7.49 ) | 5.16 ( 100.86 ) | 5.16 ( 3.81 ) | 2.37 ( 1.84 ) |
| White blood cell count decreased* | 28 | 7.18 ( 4.94 - 10.42 ) | 7.09 ( 146.7 ) | 7.09 ( 5.19 ) | 2.83 ( 2.29 ) |
| Death* | 28 | 1.9 ( 1.31 - 2.76 ) | 1.89 ( 11.82 ) | 1.89 ( 1.38 ) | 0.92 ( 0.38 ) |
| Disease progression* | 28 | 8.15 ( 5.61 - 11.83 ) | 8.05 ( 172.97 ) | 8.04 ( 5.89 ) | 3.01 ( 2.47 ) |
| Neutropenia* | 24 | 4.96 ( 3.32 - 7.42 ) | 4.92 ( 74.98 ) | 4.91 ( 3.51 ) | 2.3 ( 1.72 ) |
| Dyspnoea | 22 | 1.15 ( 0.75 - 1.74 ) | 1.14 ( 0.41 ) | 1.14 ( 0.81 ) | 0.19 ( -0.41 ) |
| Pyrexia* | 22 | 1.59 ( 1.04 - 2.42 ) | 1.58 ( 4.73 ) | 1.58 ( 1.11 ) | 0.66 ( 0.06 ) |
| Non-small cell lung cancer* | 22 | 182.43 ( 119.38 - 278.8 ) | 180.48 ( 3853.3 ) | 177.12 ( 124.21 ) | 7.47 ( 6.86 ) |
| Gamma-glutamyltransferase increased* | 20 | 19.92 ( 12.82 - 30.96 ) | 19.74 ( 355.16 ) | 19.7 ( 13.62 ) | 4.3 ( 3.67 ) |
| Abdominal pain* | 18 | 1.84 ( 1.15 - 2.92 ) | 1.83 ( 6.8 ) | 1.83 ( 1.24 ) | 0.87 ( 0.21 ) |
| Stomatitis* | 18 | 9.23 ( 5.8 - 14.68 ) | 9.15 ( 130.73 ) | 9.15 ( 6.2 ) | 3.19 ( 2.53 ) |
| Neutrophil count decreased* | 17 | 11.92 ( 7.4 - 19.22 ) | 11.83 ( 168.5 ) | 11.82 ( 7.93 ) | 3.56 ( 2.88 ) |
| Asthenia | 16 | 1.31 ( 0.8 - 2.15 ) | 1.31 ( 1.18 ) | 1.31 ( 0.87 ) | 0.39 ( -0.31 ) |
| Hyponatraemia* | 16 | 11.68 ( 7.14 - 19.11 ) | 11.6 ( 154.85 ) | 11.58 ( 7.67 ) | 3.53 ( 2.83 ) |
| Acute kidney injury* | 16 | 3.7 ( 2.26 - 6.04 ) | 3.67 ( 31.19 ) | 3.67 ( 2.43 ) | 1.88 ( 1.17 ) |
| Leukopenia* | 16 | 8.22 ( 5.03 - 13.45 ) | 8.17 ( 100.61 ) | 8.16 ( 5.4 ) | 3.03 ( 2.33 ) |
| Decreased appetite* | 15 | 2.21 ( 1.33 - 3.67 ) | 2.2 ( 9.86 ) | 2.2 ( 1.44 ) | 1.14 ( 0.41 ) |
| Blood alkaline phosphatase increased* | 15 | 14.51 ( 8.73 - 24.12 ) | 14.41 ( 186.98 ) | 14.39 ( 9.4 ) | 3.85 ( 3.12 ) |
| Muscular weakness* | 15 | 3.62 ( 2.18 - 6.02 ) | 3.61 ( 28.29 ) | 3.6 ( 2.36 ) | 1.85 ( 1.13 ) |
| Pneumonitis* | 15 | 20.07 ( 12.07 - 33.38 ) | 19.93 ( 269.31 ) | 19.89 ( 13 ) | 4.31 ( 3.59 ) |
| Neoplasm progression* | 14 | 11.19 ( 6.61 - 18.94 ) | 11.12 ( 128.88 ) | 11.11 ( 7.15 ) | 3.47 ( 2.73 ) |
| Aspartate aminotransferase increased* | 14 | 5.86 ( 3.46 - 9.92 ) | 5.83 ( 56.02 ) | 5.82 ( 3.75 ) | 2.54 ( 1.79 ) |
| Psychological trauma* | 13 | 19.53 ( 11.31 - 33.71 ) | 19.41 ( 226.61 ) | 19.37 ( 12.27 ) | 4.28 ( 3.5 ) |
| Dehydration* | 12 | 2.91 ( 1.65 - 5.13 ) | 2.9 ( 14.95 ) | 2.9 ( 1.8 ) | 1.54 ( 0.73 ) |
| Hypokalaemia* | 12 | 7.88 ( 4.47 - 13.9 ) | 7.84 ( 71.6 ) | 7.83 ( 4.87 ) | 2.97 ( 2.17 ) |
| Dermatitis* | 12 | 17.34 ( 9.82 - 30.59 ) | 17.24 ( 183.32 ) | 17.21 ( 10.7 ) | 4.11 ( 3.3 ) |
| Blood bilirubin increased* | 11 | 9.82 ( 5.43 - 17.76 ) | 9.77 ( 86.54 ) | 9.76 ( 5.94 ) | 3.29 ( 2.45 ) |
| Chronic obstructive pulmonary disease* | 11 | 9.14 ( 5.05 - 16.53 ) | 9.09 ( 79.22 ) | 9.09 ( 5.53 ) | 3.18 ( 2.35 ) |
| Hypotension | 10 | 1.49 ( 0.8 - 2.76 ) | 1.48 ( 1.58 ) | 1.48 ( 0.88 ) | 0.57 ( -0.3 ) |
| Sepsis* | 10 | 2.84 ( 1.53 - 5.29 ) | 2.83 ( 11.86 ) | 2.83 ( 1.68 ) | 1.5 ( 0.63 ) |
| Myalgia | 10 | 1.56 ( 0.84 - 2.9 ) | 1.55 ( 1.99 ) | 1.55 ( 0.92 ) | 0.64 ( -0.24 ) |
| Cerebrovascularaccident* | 10 | 2.27 ( 1.22 - 4.22 ) | 2.26 ( 7.03 ) | 2.26 ( 1.34 ) | 1.18 ( 0.3 ) |
| Urinary tract infection | 9 | 1.71 ( 0.89 - 3.29 ) | 1.71 ( 2.64 ) | 1.71 ( 0.99 ) | 0.77 ( -0.14 ) |
| Hair colour changes* | 9 | 12.77 ( 6.63 - 24.59 ) | 12.72 ( 97.06 ) | 12.7 ( 7.34 ) | 3.67 ( 2.75 ) |
| Hair disorder* | 9 | 16.01 ( 8.32 - 30.84 ) | 15.95 ( 125.91 ) | 15.92 ( 9.2 ) | 3.99 ( 3.08 ) |
| Hair texture abnormal* | 9 | 12.66 ( 6.58 - 24.38 ) | 12.61 ( 96.11 ) | 12.6 ( 7.28 ) | 3.65 ( 2.74 ) |
| Madarosis* | 9 | 14.24 ( 7.4 - 27.43 ) | 14.18 ( 110.17 ) | 14.17 ( 8.19 ) | 3.82 ( 2.91 ) |
| Hepatic function abnormal* | 9 | 6.9 ( 3.58 - 13.28 ) | 6.87 ( 45.14 ) | 6.87 ( 3.97 ) | 2.78 ( 1.86 ) |
| Hepatotoxicity* | 9 | 10.56 ( 5.48 - 20.33 ) | 10.52 ( 77.46 ) | 10.51 ( 6.07 ) | 3.39 ( 2.48 ) |
| Constipation | 9 | 1.58 ( 0.82 - 3.04 ) | 1.58 ( 1.91 ) | 1.58 ( 0.91 ) | 0.66 ( -0.26 ) |
| Pruritus | 9 | 0.72 ( 0.37 - 1.39 ) | 0.72 ( 0.98 ) | 0.72 ( 0.42 ) | -0.47 ( -1.39 ) |

Abbreviation: Asterisks (*) indicate statistically significant signals in algorithm; ROR, reporting odds ratio; PRR, proportional reporting ratio; EBGM, empirical Bayesian geometric mean; EBGM05, the lower limit of the 95% CI of EBGM; IC, information component; IC025, the lower limit of the 95% CI of the IC; CI, confidence interval; PT, preferred term.
