# Supplementary material for: Assessing the real-world safety of docetaxel for non-small cell lung cancer: Insights from a comprehensive analysis of FAERS data
Source: PLoS One. 2025 Sep 12;20(9):e0331979. doi: 10.1371/journal.pone.0331979 (PMC12431403; doi:10.1371/journal.pone.0331979)
Supplement: S8 Table — (DOCX) [file pone.0331979.s008.docx]

Supplementary Table 8:

Top 50 most frequent adverse events for Docetaxel at the PT level in patients aged over 65 from FAERS data

| PT | Case numbers | ROR(95%Cl) | PRR(χ2) | EBGM(EBGM05) | IC(IC025) |
| --- | --- | --- | --- | --- | --- |
| Diarrhoea* | 96 | 4.29 ( 3.5 - 5.27 ) | 4.11 ( 229.17 ) | 4.11 ( 3.46 ) | 2.04 ( 1.74 ) |
| Pneumonia* | 56 | 4.01 ( 3.07 - 5.23 ) | 3.91 ( 122.19 ) | 3.91 ( 3.13 ) | 1.97 ( 1.58 ) |
| Malignant neoplasm progression* | 39 | 10.16 ( 7.4 - 13.96 ) | 9.96 ( 314.58 ) | 9.95 ( 7.62 ) | 3.31 ( 2.85 ) |
| Pyrexia* | 36 | 3.54 ( 2.54 - 4.92 ) | 3.49 ( 64.18 ) | 3.49 ( 2.64 ) | 1.8 ( 1.32 ) |
| Nausea* | 35 | 1.64 ( 1.17 - 2.29 ) | 1.63 ( 8.52 ) | 1.63 ( 1.23 ) | 0.7 ( 0.21 ) |
| Dyspnoea* | 33 | 1.52 ( 1.08 - 2.14 ) | 1.51 ( 5.74 ) | 1.51 ( 1.13 ) | 0.59 ( 0.09 ) |
| Neutropenia* | 33 | 7.3 ( 5.17 - 10.3 ) | 7.18 ( 175.73 ) | 7.17 ( 5.37 ) | 2.84 ( 2.34 ) |
| Dehydration* | 33 | 5.28 ( 3.74 - 7.45 ) | 5.2 ( 112.19 ) | 5.19 ( 3.89 ) | 2.38 ( 1.88 ) |
| Febrile neutropenia* | 31 | 13.34 ( 9.35 - 19.04 ) | 13.12 ( 346.92 ) | 13.1 ( 9.73 ) | 3.71 ( 3.2 ) |
| Fatigue | 27 | 1.21 ( 0.83 - 1.77 ) | 1.2 ( 0.94 ) | 1.2 ( 0.88 ) | 0.27 ( -0.28 ) |
| Leukopenia* | 26 | 15.97 ( 10.83 - 23.53 ) | 15.75 ( 358.46 ) | 15.71 ( 11.36 ) | 3.97 ( 3.41 ) |
| Death | 25 | 0.69 ( 0.46 - 1.02 ) | 0.69 ( 3.55 ) | 0.69 ( 0.5 ) | -0.53 ( -1.1 ) |
| Decreased appetite* | 24 | 2.46 ( 1.65 - 3.68 ) | 2.44 ( 20.54 ) | 2.44 ( 1.74 ) | 1.29 ( 0.71 ) |
| Respiratory failure* | 23 | 6.77 ( 4.49 - 10.22 ) | 6.7 ( 111.59 ) | 6.69 ( 4.74 ) | 2.74 ( 2.15 ) |
| Pleural effusion* | 22 | 7.31 ( 4.8 - 11.14 ) | 7.23 ( 118.24 ) | 7.23 ( 5.08 ) | 2.85 ( 2.25 ) |
| Hypotension* | 21 | 2.36 ( 1.54 - 3.64 ) | 2.35 ( 16.34 ) | 2.35 ( 1.64 ) | 1.23 ( 0.61 ) |
| Vomiting* | 20 | 1.57 ( 1.01 - 2.43 ) | 1.56 ( 4.04 ) | 1.56 ( 1.08 ) | 0.64 ( 0.01 ) |
| Non-small cell lung cancer* | 20 | 95.63 ( 61.34 - 149.09 ) | 94.55 ( 1823.92 ) | 93.16 ( 64.25 ) | 6.54 ( 5.9 ) |
| Cardiac arrest* | 19 | 6 ( 3.82 - 9.44 ) | 5.95 ( 78.29 ) | 5.94 ( 4.07 ) | 2.57 ( 1.92 ) |
| Asthenia | 19 | 1.18 ( 0.75 - 1.86 ) | 1.18 ( 0.54 ) | 1.18 ( 0.81 ) | 0.24 ( -0.41 ) |
| White blood cell count decreased* | 18 | 4.42 ( 2.78 - 7.04 ) | 4.39 ( 47.19 ) | 4.39 ( 2.97 ) | 2.13 ( 1.47 ) |
| Stomatitis* | 17 | 8.17 ( 5.06 - 13.17 ) | 8.1 ( 105.77 ) | 8.09 ( 5.42 ) | 3.02 ( 2.33 ) |
| Mucosal inflammation* | 17 | 20.01 ( 12.4 - 32.29 ) | 19.83 ( 303.1 ) | 19.77 ( 13.25 ) | 4.31 ( 3.62 ) |
| General physical health deterioration* | 16 | 3.29 ( 2.01 - 5.38 ) | 3.27 ( 25.25 ) | 3.27 ( 2.16 ) | 1.71 ( 1 ) |
| Sepsis* | 16 | 3.42 ( 2.09 - 5.59 ) | 3.4 ( 27.12 ) | 3.4 ( 2.25 ) | 1.76 ( 1.06 ) |
| Neutrophil count decreased* | 16 | 10.05 ( 6.14 - 16.45 ) | 9.97 ( 129.04 ) | 9.96 ( 6.59 ) | 3.32 ( 2.61 ) |
| Neutropenic sepsis* | 15 | 42.67 ( 25.62 - 71.05 ) | 42.31 ( 601.09 ) | 42.03 ( 27.43 ) | 5.39 ( 4.67 ) |
| Anaemia | 14 | 1.48 ( 0.87 - 2.5 ) | 1.47 ( 2.13 ) | 1.47 ( 0.95 ) | 0.56 ( -0.19 ) |
| Septic shock* | 13 | 6.99 ( 4.05 - 12.07 ) | 6.95 ( 66.18 ) | 6.94 ( 4.4 ) | 2.8 ( 2.02 ) |
| Hypoxia* | 13 | 8.64 ( 5 - 14.91 ) | 8.58 ( 87.01 ) | 8.57 ( 5.43 ) | 3.1 ( 2.32 ) |
| Drug ineffective | 13 | 0.51 ( 0.3 - 0.88 ) | 0.52 ( 5.98 ) | 0.52 ( 0.33 ) | -0.95 ( -1.73 ) |
| Atrial fibrillation* | 13 | 2.05 ( 1.19 - 3.53 ) | 2.04 ( 6.9 ) | 2.04 ( 1.29 ) | 1.03 ( 0.25 ) |
| Pneumonitis* | 12 | 10.48 ( 5.94 - 18.5 ) | 10.41 ( 102.03 ) | 10.4 ( 6.46 ) | 3.38 ( 2.57 ) |
| Polyneuropathy* | 12 | 26.03 ( 14.73 - 45.97 ) | 25.86 ( 285.63 ) | 25.75 ( 16 ) | 4.69 ( 3.88 ) |
| Disease progression* | 11 | 2.7 ( 1.49 - 4.88 ) | 2.69 ( 11.66 ) | 2.68 ( 1.63 ) | 1.42 ( 0.59 ) |
| Dysphagia* | 11 | 3.15 ( 1.74 - 5.7 ) | 3.14 ( 16.02 ) | 3.13 ( 1.91 ) | 1.65 ( 0.81 ) |
| Acute kidney injury | 10 | 1.37 ( 0.74 - 2.55 ) | 1.37 ( 0.99 ) | 1.37 ( 0.81 ) | 0.45 ( -0.42 ) |
| Cerebral infarction* | 10 | 6.94 ( 3.73 - 12.92 ) | 6.9 ( 50.48 ) | 6.9 ( 4.1 ) | 2.79 ( 1.91 ) |
| Malaise | 9 | 0.63 ( 0.33 - 1.22 ) | 0.64 ( 1.88 ) | 0.64 ( 0.37 ) | -0.65 ( -1.57 ) |
| Hypereosinophilic syndrome* | 9 | 571.95 ( 288.61 - 1133.48 ) | 569.03 ( 4678.05 ) | 521.69 ( 294.35 ) | 9.03 ( 8.07 ) |
| Pneumothorax* | 8 | 14.47 ( 7.22 - 29.01 ) | 14.41 ( 99.64 ) | 14.38 ( 8.04 ) | 3.85 ( 2.88 ) |
| Hypokalaemia* | 8 | 3.54 ( 1.77 - 7.1 ) | 3.53 ( 14.53 ) | 3.53 ( 1.97 ) | 1.82 ( 0.86 ) |
| Urinary tract infection | 8 | 1.12 ( 0.56 - 2.24 ) | 1.12 ( 0.1 ) | 1.12 ( 0.62 ) | 0.16 ( -0.8 ) |
| Condition aggravated | 8 | 1.04 ( 0.52 - 2.08 ) | 1.04 ( 0.01 ) | 1.04 ( 0.58 ) | 0.06 ( -0.91 ) |
| Respiratory tract infection* | 8 | 10.17 ( 5.07 - 20.37 ) | 10.12 ( 65.71 ) | 10.11 ( 5.65 ) | 3.34 ( 2.37 ) |
| Cough | 8 | 0.85 ( 0.43 - 1.71 ) | 0.85 ( 0.2 ) | 0.85 ( 0.48 ) | -0.23 ( -1.19 ) |
| Weight decreased | 8 | 0.82 ( 0.41 - 1.64 ) | 0.82 ( 0.31 ) | 0.82 ( 0.46 ) | -0.28 ( -1.25 ) |
| Haemoptysis* | 8 | 6.12 ( 3.05 - 12.25 ) | 6.09 ( 34.05 ) | 6.09 ( 3.4 ) | 2.61 ( 1.64 ) |
| Confusional state | 8 | 1.04 ( 0.52 - 2.09 ) | 1.04 ( 0.01 ) | 1.04 ( 0.58 ) | 0.06 ( -0.9 ) |
| Pulmonary embolism* | 7 | 2.25 ( 1.07 - 4.73 ) | 2.25 ( 4.84 ) | 2.24 ( 1.21 ) | 1.17 ( 0.14 ) |

Abbreviation: Asterisks (*) indicate statistically significant signals in algorithm; ROR, reporting odds ratio; PRR, proportional reporting ratio; EBGM, empirical Bayesian geometric mean; EBGM05, the lower limit of the 95% CI of EBGM; IC, information component; IC025, the lower limit of the 95% CI of the IC; CI, confidence interval; PT, preferred term.
