# Supplementary material for: Assessing the real-world safety of docetaxel for non-small cell lung cancer: Insights from a comprehensive analysis of FAERS data
Source: PLoS One. 2025 Sep 12;20(9):e0331979. doi: 10.1371/journal.pone.0331979 (PMC12431403; doi:10.1371/journal.pone.0331979)
Supplement: S9 Table — (DOCX) [file pone.0331979.s009.docx]

Supplementary Table 9:

Top 50 most frequent adverse events for Docetaxel excluding common medication co-usage at the PT level from FAERS data

| PT | Case numbers | ROR(95%Cl) | PRR(χ2) | EBGM(EBGM05) | IC(IC025) |
| --- | --- | --- | --- | --- | --- |
| Diarrhoea* | 197 | 1.82 ( 1.58 - 2.11 ) | 1.79 ( 67.62 ) | 1.76 ( 1.56 ) | 0.82 ( 0.6 ) |
| Pneumonia* | 114 | 1.72 ( 1.42 - 2.08 ) | 1.7 ( 32.29 ) | 1.68 ( 1.43 ) | 0.75 ( 0.47 ) |
| Nausea* | 101 | 1.46 ( 1.19 - 1.78 ) | 1.45 ( 13.79 ) | 1.43 ( 1.21 ) | 0.52 ( 0.23 ) |
| Malignant neoplasm progression | 99 | 0.59 ( 0.48 - 0.72 ) | 0.6 ( 27.27 ) | 0.6 ( 0.51 ) | -0.73 ( -1.02 ) |
| Febrile neutropenia* | 88 | 2.88 ( 2.32 - 3.58 ) | 2.85 ( 100.67 ) | 2.75 ( 2.29 ) | 1.46 ( 1.14 ) |
| Dyspnoea* | 87 | 1.28 ( 1.04 - 1.59 ) | 1.28 ( 5.22 ) | 1.27 ( 1.06 ) | 0.35 ( 0.03 ) |
| Fatigue* | 83 | 1.33 ( 1.07 - 1.66 ) | 1.33 ( 6.65 ) | 1.32 ( 1.1 ) | 0.4 ( 0.08 ) |
| Death | 82 | 0.53 ( 0.43 - 0.66 ) | 0.54 ( 32.42 ) | 0.55 ( 0.46 ) | -0.87 ( -1.19 ) |
| Vomiting* | 74 | 1.43 ( 1.13 - 1.8 ) | 1.42 ( 9.06 ) | 1.41 ( 1.16 ) | 0.49 ( 0.15 ) |
| Dehydration* | 70 | 2.05 ( 1.61 - 2.6 ) | 2.03 ( 35.53 ) | 1.99 ( 1.63 ) | 0.99 ( 0.64 ) |
| Pyrexia | 70 | 1.23 ( 0.97 - 1.56 ) | 1.22 ( 2.83 ) | 1.22 ( 1 ) | 0.28 ( -0.06 ) |
| Neutropenia* | 70 | 2.06 ( 1.62 - 2.62 ) | 2.04 ( 36.18 ) | 2 ( 1.64 ) | 1 ( 0.65 ) |
| White blood cell count decreased* | 58 | 3.27 ( 2.5 - 4.27 ) | 3.24 ( 84.75 ) | 3.1 ( 2.48 ) | 1.63 ( 1.25 ) |
| Asthenia* | 57 | 1.4 ( 1.07 - 1.83 ) | 1.4 ( 6.28 ) | 1.39 ( 1.11 ) | 0.47 ( 0.08 ) |
| Anaemia | 51 | 0.99 ( 0.75 - 1.31 ) | 0.99 ( 0.01 ) | 0.99 ( 0.78 ) | -0.02 ( -0.42 ) |
| General physical health deterioration* | 50 | 1.84 ( 1.38 - 2.44 ) | 1.83 ( 18.24 ) | 1.8 ( 1.42 ) | 0.85 ( 0.43 ) |
| Respiratory failure* | 47 | 1.68 ( 1.25 - 2.25 ) | 1.67 ( 12.36 ) | 1.65 ( 1.29 ) | 0.72 ( 0.3 ) |
| Disease progression | 46 | 1.26 ( 0.94 - 1.69 ) | 1.26 ( 2.41 ) | 1.25 ( 0.98 ) | 0.33 ( -0.1 ) |
| Non-small cell lung cancer | 45 | 0.91 ( 0.68 - 1.23 ) | 0.91 ( 0.38 ) | 0.91 ( 0.71 ) | -0.13 ( -0.56 ) |
| Leukopenia* | 44 | 2.7 ( 1.99 - 3.66 ) | 2.68 ( 44.19 ) | 2.6 ( 2.01 ) | 1.38 ( 0.93 ) |
| Decreased appetite | 42 | 0.88 ( 0.65 - 1.19 ) | 0.88 ( 0.7 ) | 0.88 ( 0.68 ) | -0.18 ( -0.63 ) |
| Stomatitis* | 40 | 2.24 ( 1.63 - 3.08 ) | 2.23 ( 26.01 ) | 2.18 ( 1.67 ) | 1.12 ( 0.66 ) |
| Neutrophil count decreased* | 40 | 2.43 ( 1.77 - 3.35 ) | 2.42 ( 31.99 ) | 2.36 ( 1.81 ) | 1.24 ( 0.77 ) |
| Pneumonitis | 37 | 0.87 ( 0.63 - 1.21 ) | 0.88 ( 0.65 ) | 0.88 ( 0.67 ) | -0.19 ( -0.66 ) |
| Hypotension* | 35 | 2.17 ( 1.55 - 3.05 ) | 2.16 ( 21.08 ) | 2.12 ( 1.59 ) | 1.08 ( 0.59 ) |
| Pleural effusion | 32 | 0.92 ( 0.65 - 1.3 ) | 0.92 ( 0.24 ) | 0.92 ( 0.68 ) | -0.12 ( -0.63 ) |
| Haemoptysis* | 31 | 1.55 ( 1.09 - 2.22 ) | 1.55 ( 5.89 ) | 1.53 ( 1.14 ) | 0.62 ( 0.1 ) |
| Sepsis | 30 | 1.4 ( 0.97 - 2.02 ) | 1.4 ( 3.33 ) | 1.39 ( 1.02 ) | 0.47 ( -0.05 ) |
| Interstitial lung disease | 30 | 0.64 ( 0.45 - 0.92 ) | 0.65 ( 5.78 ) | 0.65 ( 0.48 ) | -0.62 ( -1.14 ) |
| Hyponatraemia* | 30 | 2.08 ( 1.44 - 3 ) | 2.07 ( 16 ) | 2.03 ( 1.49 ) | 1.02 ( 0.49 ) |
| Septic shock* | 28 | 3.56 ( 2.43 - 5.23 ) | 3.55 ( 47.98 ) | 3.38 ( 2.45 ) | 1.76 ( 1.2 ) |
| Acute kidney injury | 28 | 1.46 ( 1 - 2.13 ) | 1.46 ( 3.95 ) | 1.45 ( 1.06 ) | 0.53 ( -0.01 ) |
| Abdominal pain* | 28 | 1.59 ( 1.09 - 2.31 ) | 1.58 ( 5.83 ) | 1.56 ( 1.14 ) | 0.65 ( 0.1 ) |
| Haemoglobin decreased* | 28 | 2.04 ( 1.39 - 2.97 ) | 2.03 ( 14.1 ) | 1.99 ( 1.45 ) | 0.99 ( 0.44 ) |
| Hypokalaemia* | 27 | 2.2 ( 1.49 - 3.23 ) | 2.19 ( 16.77 ) | 2.14 ( 1.55 ) | 1.1 ( 0.54 ) |
| Cardiac arrest* | 27 | 4.08 ( 2.76 - 6.05 ) | 4.07 ( 57.85 ) | 3.84 ( 2.76 ) | 1.94 ( 1.37 ) |
| Urinary tract infection* | 25 | 2.36 ( 1.58 - 3.54 ) | 2.36 ( 18.71 ) | 2.3 ( 1.64 ) | 1.2 ( 0.62 ) |
| Cough | 22 | 0.85 ( 0.55 - 1.29 ) | 0.85 ( 0.61 ) | 0.85 ( 0.6 ) | -0.24 ( -0.84 ) |
| Neoplasm progression | 22 | 1.12 ( 0.74 - 1.72 ) | 1.12 ( 0.29 ) | 1.12 ( 0.79 ) | 0.16 ( -0.45 ) |
| Pulmonary embolism | 21 | 0.72 ( 0.47 - 1.11 ) | 0.72 ( 2.27 ) | 0.72 ( 0.5 ) | -0.47 ( -1.09 ) |
| Mucosal inflammation* | 21 | 1.75 ( 1.13 - 2.71 ) | 1.75 ( 6.52 ) | 1.72 ( 1.2 ) | 0.78 ( 0.16 ) |
| Atrial fibrillation* | 21 | 1.59 ( 1.03 - 2.46 ) | 1.59 ( 4.48 ) | 1.57 ( 1.09 ) | 0.65 ( 0.03 ) |
| Gamma-glutamyltransferase increased* | 21 | 3.05 ( 1.96 - 4.75 ) | 3.04 ( 27.23 ) | 2.93 ( 2.02 ) | 1.55 ( 0.92 ) |
| Confusional state* | 21 | 1.67 ( 1.08 - 2.58 ) | 1.67 ( 5.42 ) | 1.64 ( 1.14 ) | 0.72 ( 0.09 ) |
| Neutropenic sepsis* | 20 | 3.85 ( 2.44 - 6.07 ) | 3.84 ( 39.02 ) | 3.64 ( 2.48 ) | 1.86 ( 1.21 ) |
| Alopecia | 20 | 1.55 ( 0.99 - 2.42 ) | 1.55 ( 3.77 ) | 1.53 ( 1.05 ) | 0.61 ( -0.03 ) |
| Muscular weakness* | 19 | 2.08 ( 1.31 - 3.29 ) | 2.07 ( 10.18 ) | 2.03 ( 1.38 ) | 1.02 ( 0.36 ) |
| Fall | 19 | 1.28 ( 0.81 - 2.02 ) | 1.28 ( 1.15 ) | 1.27 ( 0.87 ) | 0.35 ( -0.3 ) |
| Hypoxia* | 18 | 1.62 ( 1.01 - 2.58 ) | 1.61 ( 4.07 ) | 1.59 ( 1.08 ) | 0.67 ( 0 ) |
| Aspartate aminotransferase increased | 18 | 1.06 ( 0.67 - 1.7 ) | 1.06 ( 0.07 ) | 1.06 ( 0.72 ) | 0.09 ( -0.58 ) |

Abbreviation: Asterisks (*) indicate statistically significant signals in algorithm; ROR, reporting odds ratio; PRR, proportional reporting ratio; EBGM, empirical Bayesian geometric mean; EBGM05, the lower limit of the 95% CI of EBGM; IC, information component; IC025, the lower limit of the 95% CI of the IC; CI, confidence interval; PT, preferred term.
